# Geometric trust-based secure localization technique for resiliency of GPS outage and location error in vehicular cyber-physical systems (VCPS)

**DOI:** 10.1038/s41598-023-48451-4

**Published:** 2023-12-15

**Authors:** Nithiyanandam N, Rajesh M, Sitharthan Ramachandran, Vengatesan K, Mohamed Imran A, Dhanamjayulu C, Baseem Khan

**Affiliations:** 1 Department of Computing Technologies, SRM Institute of Science and Technology, Kattankulathur Campus, Chengalpattu District, Kattankulathur, India; 2 Department of Computer Science Engineering, Sanjivani College of Engineering, Kopargaon, India; 3 Centre for Smart Grid Technologies, Vellore Institute of Technology, Chennai, India; 4 School of Electrical Engineering, Vellore Institute of Technology, Chennai, India; 5 School of Electrical Engineering, Vellore Institute of Technology, Vellore, Tamil Nadu India; 6 Department of Electrical and Computer Engineering, Hawassa University, Hawassa 05, Ethiopia

**Keywords:** Energy science and technology, Engineering, Mathematics and computing

## Abstract

Management of vehicle traffic is a challenging task as it is non-deterministic by nature. Vehicular Cyber-Physical Systems (VCPS) is the emerging field of dynamics of vehicle management. Vehicle localization is considered an important task in VCPS. Many researchers proposed methodologies for this based on the Global Positioning System (GPS) which poses few location identification errors. Also, there are more vulnerabilities to the existing vehicular positioning system due to Zig-Zag attacks and bad-mouth attacks. In this work, an error-free and secure environment for communication between dynamically moving vehicle models has been proposed. In our proposed model a localization technique based on mathematical geometry which is capable of GPS outages and encompasses the dynamism of vehicle and on-road trajectory has been developed. The proposed model includes Extended Kalman filter-based routing to predict the neighbouring vehicle position. To avoid vulnerabilities created by the malicious nodes, a trust-based computation is performed by each node on its neighbours perceiving the authenticity of received messages. To validate the methodology, NS_2_ tool has been used to simulate the VCPS and to test the efficiency with different scenarios such as erroneous location, GPS outage, and malicious attack. The result shows that the proposed approach is more optimal and secure than the existing methodologies.

## Introduction

**Figure 1:**
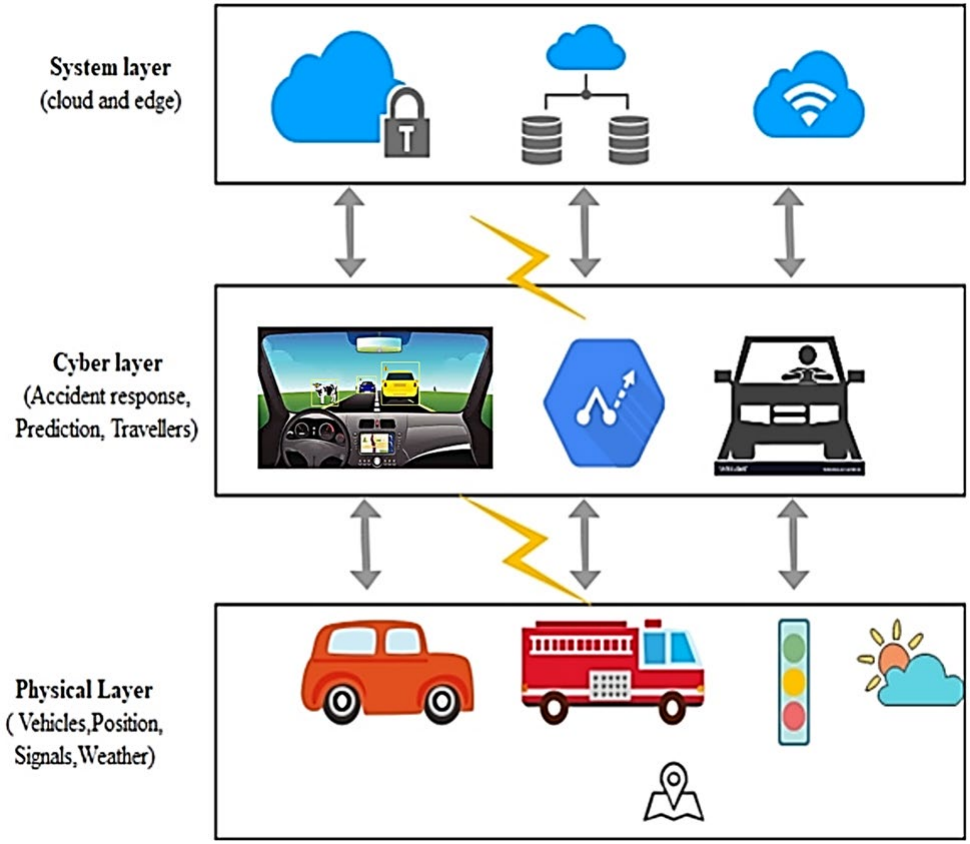
Layered approach of VCPS.

In recent decades, VCPS and the Internet of Vehicles (IoV) have increasingly combined. Also, location-based information enables Intelligent Transportation Systems (ITS) to provide enhanced safety, event response, and taxi driver assistance^1^. The layered architecture of VCPS is illustrated in Fig. 1. The physical layer comprises Vehicles, the position of vehicles, location information, and weather data. This layer is meant for data collection and sensing. The collected data are then forwarded to the cyber layer for ascertaining prediction and event responses, whereas the system layer facilitates high-performance computing by exploiting cloud services^2^.

Nowadays, vehicular localization receives increased attention among various communities, that rely on location-based services^3^. In many location-tracking applications, GPS-based localization techniques are widespread for their simplicity and cost-effectiveness. The location data have been further utilized for assessing the density of vehicles^4^, collision prediction^5^, and ascertaining Road Side Units (RSU)^6^. The accuracy of GPS localization entirely depends on signal quality and undisruptive communication throughout the VCPS network. Due to GPS outages in an ambience like high-rise buildings, fly-overs, and chaotic road trajectories, GPS-based localization services have led to performance degradation in VCPS environments^7^. It has given rise to GPS-free localization techniques in recent decades^8^. However, the deployment of these factors consumes more cost and expertise; researchers have moved their vision to identify state-of-the-art techniques to find a solution for GPS outages in VCPS environments. Besides, GPS-assisted cooperative localization, on the other hand, has provided diversified features like reduced cost, and feasibility for GPS outage problems^9^. It mainly depends on the collective communication among the vehicles and past GPS data in case of signal issues for a definite duration. Hence it requires enough location-aware vehicles and minimal speed difference. In most of the cases, the factors mentioned above are infeasible due to the following reasons. First, the target vehicle and neighbouring vehicles encountered the same outage problem in the intended location, leading to the failure to maintain the location information. Secondly, sustaining the speed difference is critical when considering urban environments. Hence, to overcome the shortcomings, the geometric localization technique is modelled in this research addressing GPS outages in VCPS environments.

Consecutively, due to the rapid proliferation of automation and sensing capabilities, traditional vehicle-based services have moved their vision to the Internet of Vehicles^10^. While considering traffic management, safe travel, and smart transportation, specific routing protocols must be addressed for accurate data dissemination. For significant information dissemination, the geographic routing protocols require the availability and scalability of the vehicles' location information. The researchers have stated that GPS receivers have paved the way for a better knowledge of location data. Nevertheless, in some cases, GPS data may mislead the end-users with erroneous positions due to signal outage, interference, and blocking^11^. Since the location information’s accuracy has the utmost importance, factors such as outdated location data, seamless mobility, and high channel overhead suppress routing protocols’ performances. Previous research has failed to address the issues like the misconception of predicted location information and location inaccuracy^12^. In this context, a novel geographic routing protocol is proposed to reduce the influence of location errors by selecting the neighbour vehicle with the least erroneous value.

Meanwhile, due to the vehicular environment’s openness, the vehicles are inevitably exposed to vulnerability posed by malicious nodes. These alarming instances are still existing in VCPS, and the combating techniques to defend the system are still sparse. Presently, several security enhancement techniques for vehicular networks have been put forward by various researchers. Out of which, trust-based models have gained attraction in recent days, due to the establishment of optimal security among the cooperative vehicular nodes^13^. Our contribution to this research is threefold.

1. First, to define a geometric localization technique for handling GPS outages in VCPS. The suggested technique assumes that each node calculates the speed, time, and direction with the utmost accuracy. Moreover, each vehicle ascertains the geometric layout for deriving the vehicle's present location in case of GPS outages. Later, relocation techniques were put forth to mitigate the effect of GPS outages in VCPS.
2. Second, to determine a novel geographic routing protocol to correct the location information using an extended Kalman filter. Here, the next forwarding vehicle is selected based on error values having the lowest score.
3. Third, to model a trust-based VCPS for detecting and isolating malicious vehicles by utilizing the data dissemination technique. Also, for analytical testing, the various attacks have been injected, and performance has been evaluated.

Recent developments in VCPS have paved the way for researchers to put forward various localization techniques for handling GPS outages. Localization techniques utilizing GPS data are used in many tracking systems to measure the network traffic and congestion on depending the speed measurements of the vehicle^14^. Likewise, it has been extended to cope with rapid urbanization by relying on minimal satellite data^15^. The work utilizes extended Kalman filter techniques to reduce location errors in vehicular networks^16^. Also, in many localization systems, RFID-based approaches are used^17^. It enriches VCPS by focusing on novel scheduling strategies for processing the messages among the nodes and junction ends. This method has been further improvised by employing kinematics-based approaches to achieve localization without using RFID. It works on the past recorded RFID data and kinematics of the vehicle to estimate the vehicle's current location^18^. Future research has begun to decrease the number of roadside units by combining distance and vehicle dynamics^19^. It is achieved by adopting the Kalman filter to reduce location errors. Most of the GPS-free localizations depend on radio communication for processing the roadside information. However, it degrades the performance at all levels by its cost and deployment of roadside units^20^. Hence, researchers have started to model GPS-free localization techniques. Grid-based localization is a prominent method that leverages data from three vehicles with stored location information and four distinct location patterns to estimate positions accurately^21^. The method reduces the deployment cost faced by GPS-assisted techniques.

Furthermore, the impact of location errors has degraded the performance of vehicular networks in recent decades. Many studies have been proposed for mitigating the effect of location error in VCPS. A behaviour analysis at the micro-level has been conducted in geographic protocols to reduce the localization techniques^22^. Maximum Expectation on Transmission Range (MER) protocol has been proposed to perform the localization in noisy environments^23^. It depends on the error probability and node information recorded in the predefined communication range. Consecutively, to reduce the location errors, Conditioned Mean Square Error Rate (CMSER) routing is designed where the decision is based on the immense distance and statistical errors^24^.

Despite the advantages, the vulnerability posed by malicious users is growing in numbers. To enhance the vehicular nodes' privacy, the reputation-dependent trust model is envisioned in^25^, where group trust is maintained among the network nodes. It serves for the resiliency against malicious attacks encountered in VCPS. Later, a fuzzy approach is proposed to enable the vehicles' decision-making process, whether to accept the particular node's messages or not. The authors in^26^ have proposed a data-centric trust model in which each node maintains an event report that entails the type of vehicle and type of event. It effectively isolates the malicious vehicle through the knowledge provided by the reports. Furthermore, the vehicle classification mechanism is discussed in^27^, in which every vehicle is reported with the nodes’ behaviour. It enables the nodes to decide with the message whether the event is trusted or malicious. Later, in^28^, a piggybacking technique is proposed in which every message is enabled with the opinion of the trustworthiness of the neighbouring nodes. This work develos a localization technique based on mathematical geometry which is capable of GPS outages and encompasses the dynamism of vehicle and on-road trajectory. The proposed model includes Extended Kalman filter-based routing to predict the neighbouring vehicle position. To avoid vulnerabilities created by the malicious nodes, a trust-based computation is performed by each node on its neighbours perceiving the authenticity of received messages. To validate the methodology, the NS2 tool has been used to simulate the VCPS and to test the efficiency with different scenarios such as erroneous location, GPS outage, and malicious attack. The result shows that the proposed approach is more optimal and secure than the existing methodologies.

The paper is organized in the following manner. The second section encompasses the “Geometric localization technique for handling GPS outages and location errors in vehicular networks”. The third section entails a “Trust-based network for security enhancement in VCPS with attacker models”. “Results and discussion” investigates the performance of the proposed system against existing security models. “Conclusion” concludes the paper.

## Geometric localization technique for handling GPS outage and location errors in vehicular networks

This section comprises two phases. First, is the Geometric Localization technique for handling GPS outages, and the next is the location prediction and correction mechanism using an Extended Kalman filter for reducing location errors.

### Geometric localization technique for handling GPS outage

In this phase, the overall design of the proposed GPS-assisted geometric localization is discussed. The suggested design assumes that all the vehicles that participated in the network can calculate distance, speed, and time. Subsequently, the vehicles have been set to communicate with each other only when they are within the transmission range. The ability of vehicles to move from one vertex to another is determined by utilizing the geographical data. Nevertheless, in many cases, the information acquired from the GPS receivers may not be accurate. Hence a location error calculation is also considered in this paper to enhance the accuracy of location information. While designing a localization mechanism resilient to GPS outage, the vehicle employs geometric model estimation to determine its location in a dynamic environment^23^.

Typically, a moving vehicle ${\text{MV}}_{{\text{x}}}$ considers its present location $({\text{a}}_{{\text{x}}} ,{\text{ b}}_{{\text{x }}} )$ by exploiting the current and statistical knowledge of the dynamism and trajectory of vehicles participated in the network. The various considerations are listed below:

- The current location information of any vehicle encompasses the direction of the vehicle ${\text{P}}_{{\text{x}}}$, Speed of vehicle ${\text{S}}_{{\text{x}}}$ and timestamp of the vehicle ${\text{TS}}_{{\text{x}}}$.
- The past location information entails the last recorded GPS location of the vehicle $\left( {{\text{a}}_{{\text{x}}}^{\prime } ,{\text{ b}}_{{\text{y}}}^{\prime } } \right)$, Direction ${\text{P}}_{{\text{x}}}^{\prime }$, Speed ${\text{S}}_{{\text{x}}}^{\prime }$ and timestamp ${\text{TS}}_{{\text{x}}}^{\prime }$.

Let us consider a scenario of a GPS outage with a long duration on a curved road. It exploits the vehicles’ previously recorded GPS location data and the present location of the intended vehicle. Specifically, the road sections other than meeting at 0 and 180 degrees are considered divergent. In this case, the current and previous locations of the vehicles may be different.

1 $${{\rm {P}}_{\rm {x}}} \ne {{ {\rm P}^{\prime }}_{\rm {x}}}$$

**Figure 2:**
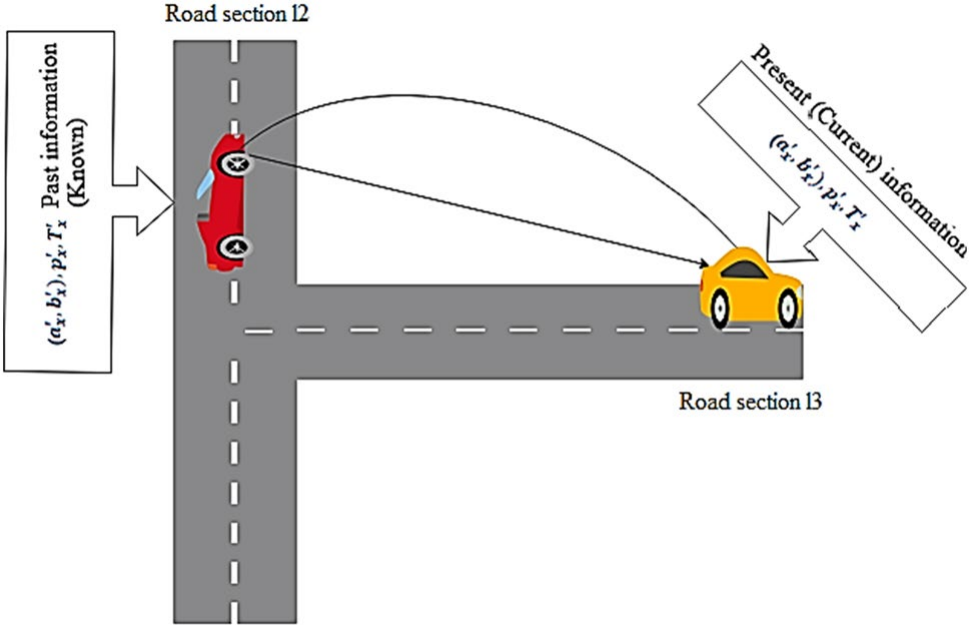
GPS outage in curved road.

While the vehicle ${\text{MV}}_{{\text{x}}}$ is receiving the GPS signals in mobility, the value of $\left| {{\text{P}}_{{\text{x}}} - {{\text P}^{\prime }}_{{\text{x}}}} \right| \ne 180^{ \circ }$. Consider when the vehicle travels in a curved road, it faces the GPS outage problem, as depicted in Fig. 2.

Here, the past information comprises known data and current information contains unknown data. To mitigate the problem, the geometric layout is modelled in this paper. Accordingly, the distance covered by the vehicle ${\mathrm{MV}}_{\mathrm{x}}$ on the straight road section and curved section is given in Eqs. (2) and (3)

2 $${\text{P}}_{{\text{x}}} = \frac{{\left( {{\text{TS}}_{{\text{x}}} - {\text{TS}}_{{\text{x}}}^{\prime } } \right)}}{2}{ } \times {\text{ S}}_{{\text{x}}}^{\prime }$$

3 $${\text{P}}_{{\text{x}}} = \frac{{\left( {{\text{TS}}_{{\text{x}}} - {\text{TS}}_{{\text{x}}}^{\prime } } \right)}}{2}{ } \times {\text{ S}}_{{\text{x}}}$$

The geometric model includes the circles C_2_ and C_3_ and the road sections RS_1_ and RS_2_. RS_3_ is another road line that passes through the center of RS_2_ at an angle ${\text{C}\kern-2.1pt\raisebox{1.25pt}{\reflectbox{'}}}$_2_. Known data: center of the circle $\left( {{\text{a}}_{{\text{x}}}^{\prime } ,{\text{ b}}_{{\text{y}}}^{\prime } } \right)$ and the point $\left( {{\text{a}}_{{\text{x}}}^{\prime } ,{\text{ b}}_{{\text{y}}}^{\prime } } \right)$, on RS_3_. The angle ${\text{C}\kern-2.1pt\raisebox{1.25pt}{\reflectbox{'}}}$ = $\frac{{{\text{P}}_{{\text{x}}} }}{{{\text{radius}}}}$ where radius of RS_3_ is obtained from the trajectory and the arc of RS_3_. Likewise, the distance ${\text{P}}$ of the previously identified location $\left( {{\text{a}}_{{\text{x}}}^{\prime } ,{\text{ b}}_{{\text{y}}}^{\prime } } \right)$ and the present location $({\text{a}}_{{\text{x}}} ,{\text{ b}}_{{\text{x }}} )$ at angle ${\text{C}\kern-2.1pt\raisebox{1.25pt}{\reflectbox{'}}}$_1_ is given in Eq. (4).

4 $${\text{P}} = \sqrt {{\text{P}}_{1}^{2} + {\text{P}}_{3}^{2} - 2{\text{P}}_{1} {\text{P}}_{3} \cos {\text{C}\kern-2.1pt\raisebox{1.25pt}{\reflectbox{'}}}_{1}}$$

where ${\text{P}}_{1}$ and ${\text{P}}_{3}$ are the distances that are already known. The equation of circle C_2_ with the known values of P and location $\left( {{\text{a}}_{{\text{x}}}^{\prime } ,{\text{ b}}_{{\text{y}}}^{\prime } } \right)$ is given in Eq. (5).

5 $${\text{P}}^{2} = \left( {{\text{a}} - {\text{a}}_{{\text{x}}}^{\prime } } \right)^{2} + \left( {{\text{b}} - {\text{b}}_{{\text{x}}}^{\prime } } \right)^{2}$$

The quadratic equation’s obtained from the Eq. (3) and Eq. (4) is expressed in Eq. (6) and Eq. (7).

6 $${\text{a}}^{2} - 2{\text{aa}}_{{\text{x}}}^{\prime } + \left( {{\text{a}}_{{\text{x}}}^{\prime } } \right) - \frac{{{\text{P}}^{2} }}{{1 + \left( {{\text{P}}_{{\text{x}}}^{\prime } } \right)^{2} }}$$

7 $${\text{b}}^{2} - 2{\text{bb}}_{{\text{x}}}^{\prime } + \left( {{\text{b}}_{{\text{x}}}^{\prime } } \right) - \frac{{{\text{P}}^{2} \left( {{\text{P}}_{{\text{x}}}^{\prime } } \right)^{2} }}{{1 + \left( {{\text{P}}_{{\text{x}}}^{\prime } } \right)^{2} }}$$

The location of the vehicle (a,b) is then obtained by the solving Eqs. (6) and (7)

8 $$\left( {{\text{a}},{\text{b}}} \right) = \left( {{\text{a}}_{{\text{x}}}^{\prime } \pm \frac{{\text{P}}}{{\sqrt {1 + \left( {{\text{P}}_{{\text{x}}}^{\prime } } \right)^{2} } }},{\text{ b}}_{{\text{x}}}^{\prime } \pm \frac{{{\text{P}} \times \left( {{\text{P}}_{{\text{x}}}^{\prime } } \right)^{2} }}{{\sqrt {1 + \left( {{\text{P}}_{{\text{x}}}^{\prime } } \right)^{2} } }}} \right)$$

Furthermore, the present location $({\text{a}}_{{\text{x}}} ,{\text{ b}}_{{\text{x }}} )$ of the vehicle is usually lies at the center of the circle C_2_. Hence, the location value as per Eq. (4) is illustrated in Eq. (9).

9 $${\text{P}}^{2} = \left( {{\text{a}}_{{\text{x}}} - {\text{a}}_{{\text{x}}}^{\prime } } \right)^{2} + \left( {{\text{b}}_{{\text{x}}} - {\text{b}}_{{\text{x}}}^{\prime } } \right)^{2}$$

Hence, the present location of the vehicle is determined by Eq. (9).

10 $$({\text{a}}_{{\text{x}}} ,{\text{ b}}_{{\text{x }}} ) = {\text{a}}_{{\text{x}}}^{\prime } \pm \left( {\begin{array}{*{20}c} {\sqrt {1 \pm \left( {1 + {\text{p}}_{{\text{x}}}^{2} } \right){\text{T}}^{2} - \left( {1 + {\text{a}}_{{\text{x}}}^{2} } \right){\text{P}}^{2} } ,} \\ {{\text{b}}_{{\text{x}}}^{\prime } \pm \sqrt {1 \pm \left( {1 + {\text{p}}_{{\text{x}}}^{\prime } } \right){\text{T}}^{2} - \left( {1 + {\text{a}}_{{\text{x}}}^{\prime } } \right){\text{P}}^{2} } } \\ \end{array} } \right)$$

where, ${\text{T}} = \frac{{{\text{P}} \times {\text{p}}_{{\text{x}}} }}{{\sqrt {1 + {\text{p}}_{{\text{x}}}^{2} } }} \mp \frac{{{\text{P}} \times {\text{p}}_{{\text{x}}}^{2} }}{{\sqrt {1 + {\text{p}}_{{\text{x}}}^{2} } }}$ yields a constant value. After determining the location, the impact of GPS outages should be reduced in vehicular networks. Dynamic relocation technique is utilized to accomplish this task by selecting an intermediate vehicle. Accordingly, the vehicles that lie within the communication range of the intermediate node are considered. Later, for ascertaining the location-based services, a random vehicle is selected as an intermediary based on the dynamism recorded for that particular vehicle. It depends on the following measures,

$${\text{P}}_{{\text{x}}}$$:
Present direction of the vehicle.
$${\text{S}}_{{\text{x}}}$$ :
Speed of the vehicle recorded.
$${\text{ND}}_{{{\text{P}}_{{\text{x}}} }}$$ :
Total number of changes in direction.
$${\text{ND}}_{{{\text{S}}_{{\text{x}}} }}$$ :
Total number of speed change.

During the start of the communication, the intermediate vehicle transmits the relocation request to its neighboring vehicle. Once received the request, the neighboring vehicle acknowledges it with the dynamism information. Then the cumulative weight ${\mathbb{\Gamma}}_{{\text{R}}}$ of the relocation information for each vehicle is calculated and the vehicle that having the maximum weight is considered for dynamic relocation. The weight of the change in direction and speed is expressed in Eqs. (11) and (12).11

12 $${\mathbb{\Gamma}}_{{{\text{ND}}_{{{\text{S}}_{{\text{x}}} { }}} }} = 1 - {{ \lambda }}^{{{\text{ND}}_{{{\text{S}}_{{\text{x}}} { }}} }}$$

where € is the decisive factor of direction. $\uplambda$ is the decisive factor of speed. The cumulative weight is ${\mathbb{\Gamma}}_{{\text{R}}}$ is expressed in (13).

13 $${\mathbb{\Gamma}}_{{\text{R}}} = {\mathbb{\Gamma}}_{{{\text{ND}}_{{{\text{P}}_{{\text{x}}} { }}} }} + {\mathbb{\Gamma}}_{{{\text{ND}}_{{{\text{P}}_{{\text{x}}} }} }}$$

### Location prediction and correction mechanism using an Extended Kalman filter for reducing location errors

After reducing the impact of the GPS outages, performance degradation caused by location errors is considered. In recent decades, the location error in measurement leads to inaccuracy of the predicted information about the future location of the nodes. To overcome the shortcomings, Extended Kalman filter is used for correction and prediction of the location information. Initially, the geographic location of the vehicles is acquired from the GPS receivers. The relation between the real value ${\text{a}}_{{\text{x}}} \left( {\text{T}} \right)$ and measured value ${\text{a}}_{{\text{x}}}^{\prime } \left( {\text{T}} \right)$ of the vehicle ${\text{MV}}_{{\text{x}}}$ at the time T is given by Eq. (14).

14 $${\text{a}}_{{\text{x}}}^{\prime } \left( {\text{T}} \right) = {\text{a}}_{{\text{x}}} \left( {\text{T}} \right) + { }\varepsilon \left( {\text{T}} \right)$$

$\varepsilon \left( {\text{T}} \right)$ is the location error occurred in measurement. Likewise, the future location of the vehicle for the succeeding timestamp is given as in Eq. (15).

15 $$\widehat{{{\text{a}}_{{\text{x}}}^{\prime } }}\left( {{\text{T}} + \phi {\text{T}}} \right) = {\text{ a}}_{{\text{x}}}^{\prime } \left( {\text{T}} \right) + {\text{MV}}_{{\text{k}}} \left( {\text{T}} \right){* }\phi {\text{T}}$$

**Figure 3:**
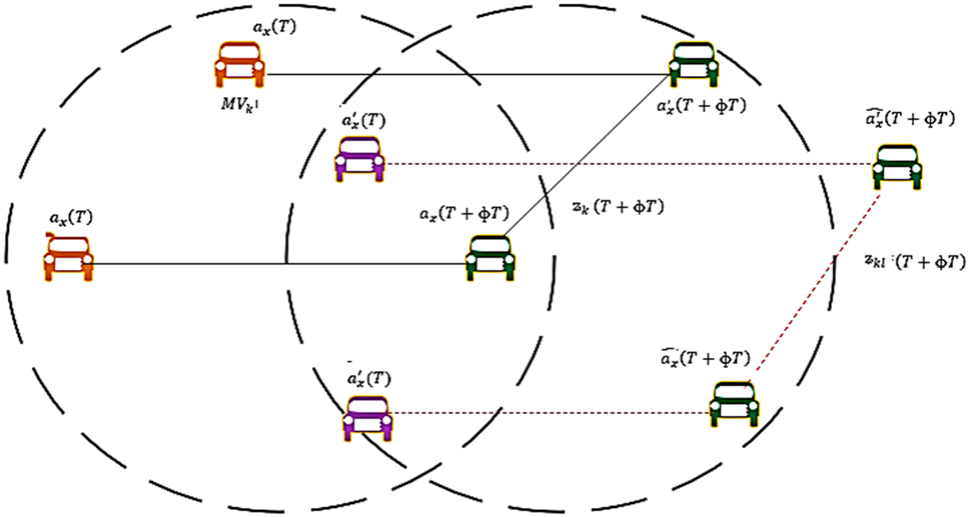
Future location of vehicles.

As illustrated in Fig. 3, the futurity location of the vehicle ${\text{MV}}_{{\text{k}}} {\text{ and MV}}_{{\text{l}}}$ are obtained from ${\text{a}}_{{\text{x}}}^{\prime } \left( {\text{T}} \right)$ and ${\text{a}}_{{\text{x}}} \left( {\text{T}} \right)$ respectively. The GPS receivers however poses some localization errors of the range − 10 to + 10 for a distance of 50 m. Subsequently, the location error of the vehicle ${\text{MV}}_{{\text{k}}}$ is calculated per Eq. (15). It is the difference between real location and estimated location.16

And, the actual distance  from ${\text{MV}}_{{\text{k}}}$ to ${\text{MV}}_{{\text{l}}}$ is given in Eq. (17).17

**Figure 4:**
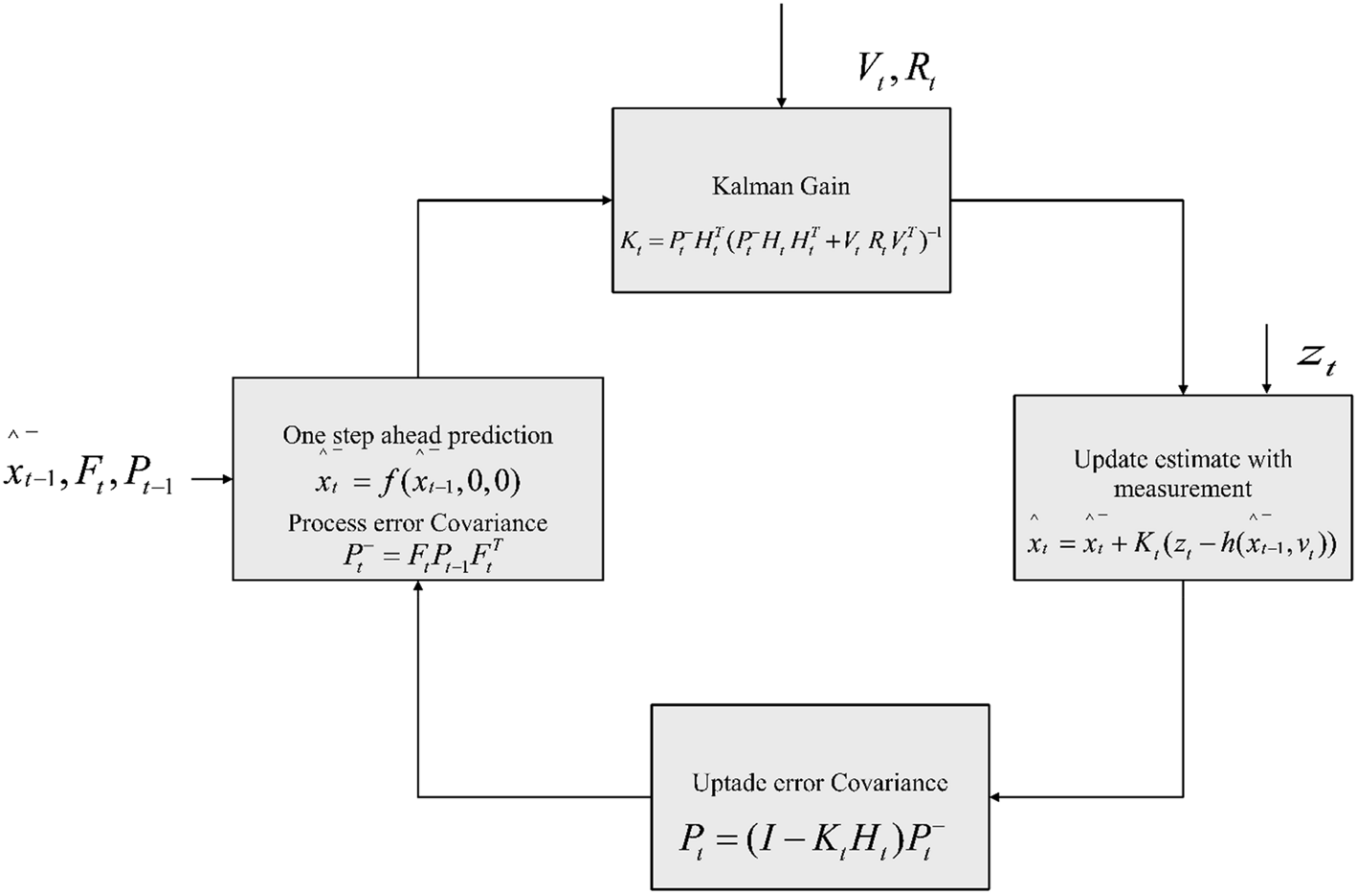
Process of Extended Kalman Filter.

For predicting and correcting the errors in the considered non-linear vehicular network, Extended Kalman Filter (EKF)^26^ is designed. The overall process of the EKF is illustrated in Fig. 4. The framework for event detection is shown where how the EKF function and their operation is visualized. According to EKF, the process model of the system is defined in Eq. (18), whereas, the measurement model is given in Eq. (19).

18 $$\overline{{\widehat{{{\text{a}}_{{\text{t}}} }}}} = {\text{f}}\left( {\overline{{\widehat{{{\text{a}}_{{{\text{t}} - 1}} }}}} ,{\text{ I}}_{{\text{t}}} ,{\text{n}}_{{\text{t}}} } \right)$$

19 $${\text{L}}_{{\text{t}}} = {\text{h}}\left( {\widehat{{{\text{a}}_{{{\text{t}} - 1}} }},{\text{ b}}_{{\text{t}}} } \right)$$

20 $${\text{M}}_{{\text{T}}} = \left[ {\begin{array}{*{20}c} 1 \\ 0 \\ 0 \\ 0 \\ 0 \\ 0 \\ \end{array} { }\begin{array}{*{20}c} {0 } \\ 1 \\ 0 \\ 0 \\ 0 \\ 0 \\ \end{array} \begin{array}{*{20}c} { \Delta t} \\ 0 \\ 1 \\ 0 \\ 0 \\ 0 \\ \end{array} \begin{array}{*{20}c} 0 \\ {\Delta t} \\ 0 \\ 1 \\ 0 \\ 0 \\ \end{array} \begin{array}{*{20}c} { 0 } \\ 0 \\ 0 \\ 0 \\ 1 \\ 0 \\ \end{array} \begin{array}{*{20}c} 0 \\ 0 \\ 0 \\ 0 \\ 0 \\ 1 \\ \end{array} } \right]$$

$\overline{{\widehat{{{\text{a}}_{{{\text{t}} - 1}} }}}}$ is the location of the vehicle at time t−1, ${\text{I}}_{{\text{t}}}$ is the input given to the vehicle at time t, ${\text{n}}_{{\text{t}}}$ is the noise processed at time t, and ${\text{b}}_{{\text{t}}}$ is the noise measured at time t.

To predict the node in the non-linear vehicular network, the filter must be initiated with the following parameters, ${\text{M}}_{{\text{T}}} ,{\text{ U}}_{{\text{T}}} ,{\text{ R}}_{{\text{T}}} ,{\text{J}}_{{\text{T}}} ,{\text{ K}}_{{\text{T}}} ,{\text{V}}_{{\text{T}}} ,{\text{ and O}}_{{\text{T}}}$. Where, ${\text{M}}_{{\text{T}}}$ is the Jacobian matrix which is obtained from ${\text{f}}\left( {\overline{{\widehat{{{\text{a}}_{{{\text{t}} - 1}} }}}} ,{\text{ I}}_{{\text{t}}} ,{\text{n}}_{{\text{t}}} } \right)$ at the time t−1. $\Delta {\text{t}}$ is the assumed sampling time intervals. The initial value of ${\text{M}}_{{\text{T}}}$ is given in the below matrix Eq. (20). Whereas, the initial value of the process noise ${\text{U}}_{{\text{T}}}$ is given in the below zero matrix (Eq. 21), because the process noise is assumed to be zero initially.

21 $${\text{U}}_{{\text{T}}} = { }\left[ {\begin{array}{*{20}c} 0 \\ 0 \\ 0 \\ 0 \\ 0 \\ 0 \\ \end{array} { }\begin{array}{*{20}c} 0 \\ 0 \\ 0 \\ 0 \\ 0 \\ 0 \\ \end{array} { }\begin{array}{*{20}c} 0 \\ 0 \\ 0 \\ 0 \\ 0 \\ 0 \\ \end{array} { }\begin{array}{*{20}c} { 0 } \\ 0 \\ 0 \\ 0 \\ 0 \\ 0 \\ \end{array} { }\begin{array}{*{20}c} 0 \\ 0 \\ { 0 } \\ 0 \\ 0 \\ 0 \\ \end{array} { }\begin{array}{*{20}c} 0 \\ 0 \\ 0 \\ 0 \\ 0 \\ 0 \\ \end{array} } \right]$$

Then the measurement matrix ${\text{R}}_{{\text{T}}}$ and measurement noise ${\text{J}}_{{\text{T}}}$ must be found. The Jacobian matrix of ${\text{R}}_{{\text{T}}} {\text{ and J}}_{{\text{T}}}$ is given in Eqs. (22) and (23), respectively.

22 $${\text{R}}_{{\text{T}}} = { }\left[ {\begin{array}{*{20}c} 1 \\ 0 \\ 0 \\ 0 \\ 0 \\ 0 \\ \end{array} { }\begin{array}{*{20}c} 0 \\ 1 \\ 0 \\ 0 \\ 0 \\ 0 \\ \end{array} { }\begin{array}{*{20}c} 0 \\ 0 \\ 1 \\ 0 \\ 0 \\ 0 \\ \end{array} { }\begin{array}{*{20}c} { 0 } \\ 0 \\ 0 \\ 1 \\ 0 \\ 0 \\ \end{array} { }\begin{array}{*{20}c} 0 \\ 0 \\ { 0 } \\ 0 \\ 1 \\ 0 \\ \end{array} { }\begin{array}{*{20}c} 0 \\ 0 \\ 0 \\ 0 \\ 0 \\ 1 \\ \end{array} } \right]$$

Finally, the process noise ${\text{O}}_{{\text{T}}}$ is the error observed in the computation. It is then set as minimum to design an error free vehicular network as shown in Eq. (23).

23 $${\text{O}}_{{\text{T}}} = \left[ {\begin{array}{*{20}c} {0.1} \\ 0 \\ 0 \\ 0 \\ 0 \\ 0 \\ \end{array} \begin{array}{*{20}c} 0 \\ {0.1} \\ 0 \\ 0 \\ 0 \\ 0 \\ \end{array} \begin{array}{*{20}c} 0 \\ 0 \\ 0 \\ {0.1} \\ 0 \\ 0 \\ \end{array} \begin{array}{*{20}c} 0 \\ 0 \\ 0 \\ 0 \\ {0.1} \\ 0 \\ \end{array} \begin{array}{*{20}c} 0 \\ 0 \\ 0 \\ 0 \\ 0 \\ {0.1} \\ \end{array} } \right]$$

For attaining the best estimation, the values ${\text{V}}_{{\text{T}}}$ and ${\text{O}}_{{\text{T}}}$ can be tuned further to minimize the location errors. ${\text{R}}_{{\text{T}}}$ and ${\text{J}}_{{\text{T}}}$ are obtained by differentiating the function ${\text{h}}\left( {\widehat{{{\text{a}}_{{{\text{t}} - 1}} }},{\text{ b}}_{{\text{t}}} } \right)$. with respect to time t−1.

24 $${\text{J}}_{{\text{T}}} = { }\left[ {\begin{array}{*{20}c} 1 \\ 0 \\ 0 \\ 0 \\ 0 \\ 0 \\ \end{array} { }\begin{array}{*{20}c} 0 \\ 1 \\ 0 \\ 0 \\ 0 \\ 0 \\ \end{array} { }\begin{array}{*{20}c} 0 \\ 0 \\ 1 \\ 0 \\ 0 \\ 0 \\ \end{array} { }\begin{array}{*{20}c} { 0 } \\ 0 \\ 0 \\ 1 \\ 0 \\ 0 \\ \end{array} { }\begin{array}{*{20}c} 0 \\ 0 \\ { 0 } \\ 0 \\ 1 \\ 0 \\ \end{array} { }\begin{array}{*{20}c} 0 \\ 0 \\ 0 \\ 0 \\ 0 \\ 1 \\ \end{array} } \right]$$

The error covariance matrix obtained by the filter at the time T is given in Eq. (24). The following are the assumptions made:

- The diagonal elements represent the covariance in the state of the vehicles.
- The non-diagonal elements denote the independent values representing the correlation. And, it has no longer effect on the matrix values.
  25 $${\mathrm{V}}_{\mathrm{T}}=\left[\begin{array}{c}e\left(\mathrm{a},\mathrm{a}\right)\\ e\left(\mathrm{b},\mathrm{a}\right)\\ e\left(\mathrm{n},\mathrm{a}\right)\\ e\left(\mathrm{n},\mathrm{a}\right)\\ e\left(\mathrm{x},\mathrm{ a}\right)\\ e\left(\mathrm{y},\mathrm{a}\right)\end{array} \begin{array}{c}e\left(\mathrm{a},\mathrm{b}\right)\\ e\left(\mathrm{b},\mathrm{b}\right)\\ e\left(\mathrm{n},\mathrm{b}\right)\\ e\left(\mathrm{n},\mathrm{b}\right)\\ e\left(\mathrm{a},\mathrm{b}\right)\\ e\left(\mathrm{y},\mathrm{b}\right)\end{array} \begin{array}{c}e\left(\mathrm{a},\mathrm{n}\right)\\ e\left(\mathrm{b},\mathrm{n}\right)\\ e\left(\mathrm{n},\mathrm{n}\right)\\ e\left(\mathrm{x},\mathrm{n}\right)\\ e\left(\mathrm{y},\mathrm{n}\right)\\ e\left(\mathrm{a},\mathrm{n}\right)\end{array} \begin{array}{c}e\left(\mathrm{a},\mathrm{y}\right)\\ e\left(\mathrm{b},\mathrm{y}\right)\\ e\left(\mathrm{x},\mathrm{y}\right)\\ e\left(\mathrm{y},\mathrm{y}\right)\\ e\left(\mathrm{a},\mathrm{y}\right)\\ e\left(\mathrm{n},\mathrm{y}\right)\end{array} \begin{array}{c}e\left(\mathrm{a},\mathrm{x}\right)\\ e\left(\mathrm{b},\mathrm{x}\right)\\ e\left(\mathrm{x},\mathrm{a}\right)\\ e\left(\mathrm{n},\mathrm{a}\right)\\ e\left(\mathrm{x},\mathrm{x}\right)\\ e\left(\mathrm{x},\mathrm{y}\right)\end{array} \begin{array}{c}e\left(\mathrm{a},\mathrm{y}\right)\\ e\left(\mathrm{b},\mathrm{y}\right)\\ e\left(\mathrm{n},\mathrm{y}\right)\\ e\left(\mathrm{a},\mathrm{b}\right)\\ e\left(\mathrm{b},\mathrm{b}\right)\\ e\left(\mathrm{y},\mathrm{y}\right)\end{array}\right]$$

Eventually, the error covariance matrix is given in Eq. (26). It is assumed that the ${\mathrm{V}}_{0}$ is set to maximum for minimizing the gap in error.

26 $${\text{V}}_{0} = \left[ {\begin{array}{*{20}c} {1000} \\ 0 \\ 0 \\ 0 \\ 0 \\ 0 \\ \end{array} \begin{array}{*{20}c} 0 \\ {1000} \\ 0 \\ 0 \\ 0 \\ 0 \\ \end{array} \begin{array}{*{20}c} 0 \\ 0 \\ {1000} \\ 0 \\ 0 \\ 0 \\ \end{array} \begin{array}{*{20}c} 0 \\ 0 \\ 0 \\ {1000} \\ 0 \\ 0 \\ \end{array} \begin{array}{*{20}c} 0 \\ 0 \\ 0 \\ 0 \\ {1000} \\ 0 \\ \end{array} \begin{array}{*{20}c} 0 \\ 0 \\ 0 \\ 0 \\ 0 \\ {1000} \\ \end{array} } \right]$$

Meanwhile, the measurement noise ${\text{O}}_{{\text{T}}}$ is given in Eq. (27), Where ${\text{O}}_{{\text{a}}}$ and ${\text{O}}_{{\text{b}}}$ is the latitudinal and longitudinal changes respectively.

27 $${\text{O}}_{{\text{T}}} = \left[ {\begin{array}{*{20}c} {{\text{J}}_{{\text{a}}} } \\ 0 \\ \end{array} { }\begin{array}{*{20}c} 0 \\ {{\text{J}}_{{\text{b}}} } \\ \end{array} } \right]$$

The initial value of the measurement noise ${\text{J}}_{{\text{T}}}$ is given in Eq. (25).

28 $${\text{O}}_{{\text{T}}} = \left[ {\begin{array}{*{20}c} 1 \\ 0 \\ \end{array} { }\begin{array}{*{20}c} 0 \\ 1 \\ \end{array} } \right]$$

## Trust-based model for attacker detection in VCPS

**Figure 5:**
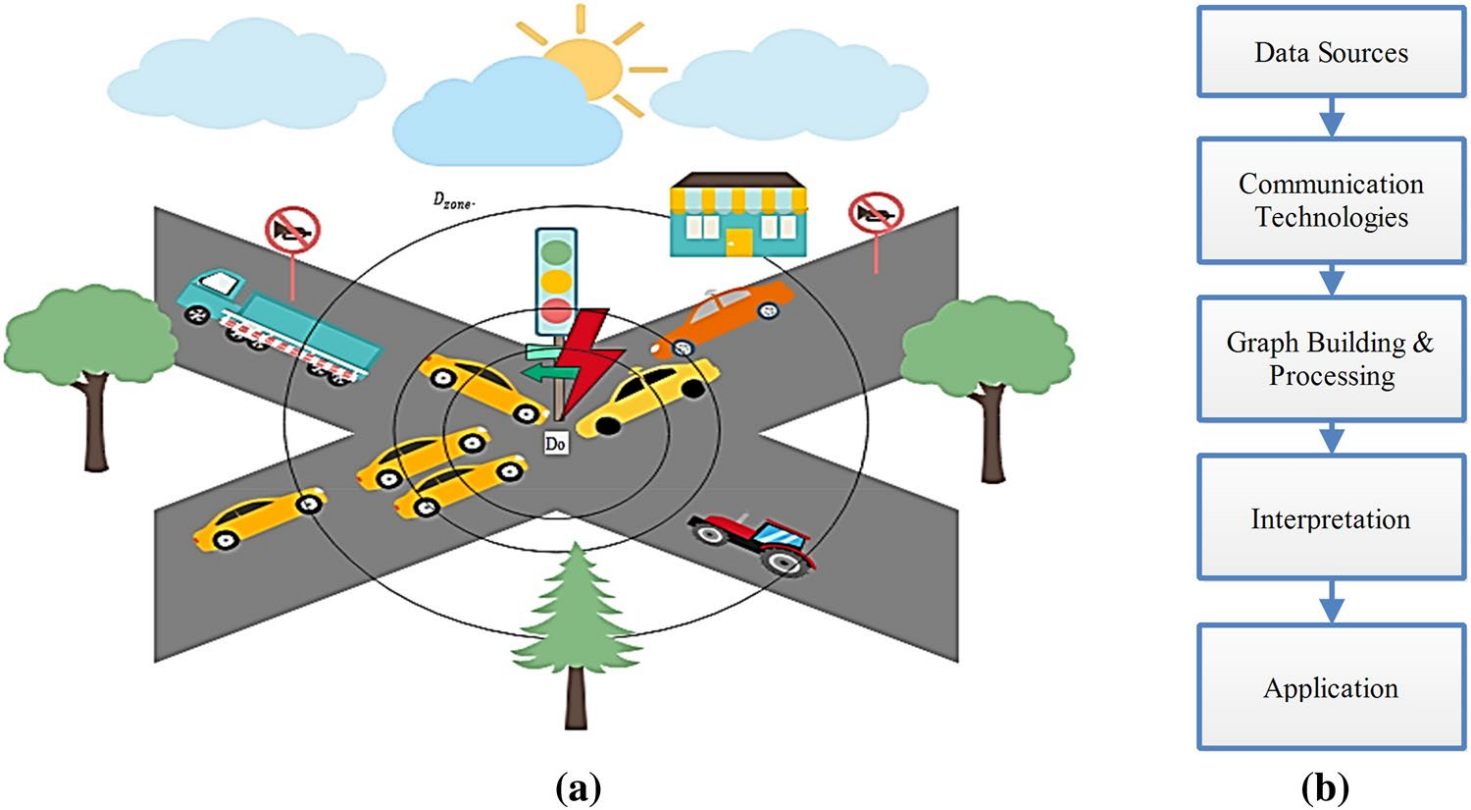
(**a**) Event Detection in VCPS. (**b**) Flow chart VCPS.

In most of the VCPS, the vehicles are involved in seamless data transfer to exchange data about cautious events and accident zones as shown in Fig. 5a. In most cases, these vehicular networks may be encountered with fraudulent events such as data tampering, disruptive communication, and information modification with noise. Figure 5b shows the flowchart of the VCPS. Hence, in addition to the geometric localization and location error prediction, the security of the VCPS is also addressed in this research. To achieve reliability and availability in VCPS, a trust-based model is developed to detect the malicious vehicle by utilizing data dissemination techniques some model uses flying Ad-hoc networks for reliability^27^. In our proposed model, the large scale prototyped vehicular network generates a set of events. The number of vehicles that are intended to create the set of events is denoted as ${\text{N}}_{{\text{E}}}$.For every event E, the authentication is performed by exchanging two values 0 and 1 denoted by ${\text{E}}_{{{\text{trust}}}}$.

$${\text{E}}_{{{\text{trust}}}}$$= 1 $$\left\{ {\text{event with both malicious and benevolent nodes}} \right\}$$

When ${\text{E}}_{{{\text{trust}}}}$= 1 event is considered as a trusted communication occurred on the road.

$${\text{E}}_{{{\text{trust}}}}$$ = 0 $$\left\{ {\text{event with only malicious nodes}} \right\}$$

Alternatively, when ${\text{E}}_{{{\text{trust}}}}$= 0, it is untrusted communication occurred among the vehicles in the network. Here, authentic event comprises of accident alert, traffic signs, and curved paths. And, Malicious event are the messages exchanged by the vulnerable nodes deliberately across the vehicular networks.

Consider, a geographic area consists of a Zone meant for detection. ${\text{D}}_{{{\text{zone}}}}$ encompasses the coordinates (x,y) and the radius r. In our system, all the vehicles that are lies in the ${\text{D}}_{{{\text{zone}}}}$ are informed with the authentic and malicious events stated above. It is assumed that the trusted event E having ${\text{E}}_{{{\text{trust}}}}$= 1 is observed by all nodes with real events lies in ${\text{D}}_{{{\text{zone}}}}$. Whereas, the untrusted event E with ${\text{E}}_{{{\text{trust}}}}$= 0 is sensed only by the malicious nodes lies within ${\text{D}}_{{{\text{zone}}}}$. When the event is detected, the vehicles in the network broadcast the alert messages ${\text{M}}_{{{\text{alert}}}}$ among its neighboring nodes in ${\text{D}}_{{{\text{zone}}}} .$

The proposed model exhibits two misbehavior aspects:

1. The malevolent node ${\text{M}}_{{\text{N}}}$ alters the ${\text{E}}_{{{\text{trust}}}}$ with the probability of ${\text{P}}_{{{\text{MN}}}}$. All the vehicles in the network posses the ${\text{P}}_{{{\text{MN}}}}$ ranges from 0 to 1.
2. Broadcasting of fake, fictitious events by the malevolent nodes across the network.

During an event, the ${\text{M}}_{{{\text{alert}}}}$ are generated in ${\text{D}}_{0}$ are gets progressed throughout the ${\text{D}}_{{{\text{zone}}}}$ as depicted in Fig. 4. Here, the data dissemination starts from ${\text{D}}_{0}$ to ${\text{D}}_{{{\text{zone}}}}$. The yellow cars represent the trusted nodes and red color vehicle denotes the malicious vehicle.

When the vehicles ${\text{N}}_{{\text{E}}}$ detects the event E in the ${\text{D}}_{0}$ region, it starts broadcasting the warning messages across the network. The authentic warning message consists of the following parameters as in Eq. (28).

29 $${\text{W}}_{{{\text{message}}}} = \left\langle {{\text{E}}_{{{\text{ID}}}} ,{\text{a}},{\text{b}},{\text{ T}}_{{\text{i}}} ,{\text{E}}_{{{\text{trust}}}} ,{\text{ seqnum}}} \right\rangle$$

Here ${\text{E}}_{{{\text{ID}}}}$ is the unique ID of the event E, (a,b) is the position of the vehicles at the instance ${\text{T}}_{{\text{i}}}$, ${\text{seqnum}}$ is the message’ sequence number. When detecting the ${\text{W}}_{{{\text{message}}}}$ for an event E, two functionalities have been performed.

1. If ${\text{E}}_{{{\text{trust}}}}$= 1, the vehicle broadcast ${\text{M}}_{{{\text{alert}}}}$ to all the nodes lies within ${\text{D}}_{{{\text{zone}}}}$. The data format of the ${\text{M}}_{{{\text{alert}}}}$ is illustrated in Eq. (30).
  30 $${\text{M}}_{{{\text{alert}}}} = \left\langle {{\text{E}}_{{{\text{ID}}}} ,{\text{a}},{\text{b}},{\text{ T}}_{{\text{i}}} ,{\text{Trust}},{\text{ seqnum}}} \right\rangle$$

The value ${\text{Trust}}$ is set by analyzing the vehicles’ behavior. When the vehicle is malicious then it is set as 0 with the probability of ${\text{P}}_{{{\text{MN}}}}$. And, the value of ${\text{Trust}}$ is 1 if no such malicious event detected. The processing of ${\text{W}}_{{{\text{message}}}}$ constitutes the following steps:

- When the benevolent(trusted) nodes detect the ${\text{W}}_{{{\text{message}}}}$ it checks for the ${\text{E}}_{{{\text{trust}}}}$
- If ${\text{E}}_{{{\text{trust}}}}$= 1 it sets the value of ${\text{Trust}}$ to 1.
- Otherwise it neglects the ${\text{W}}_{{{\text{message}}}}$
- Finally, it sends ${\text{M}}_{{{\text{alert}}}}$ to all nodes in detection zone.

2. If ${\text{E}}_{{{\text{trust}}}}$= 0, indicates that the event is malicious. Subsequently, the vehicle broadcast the ${\text{M}}_{{{\text{alert}}}}$ only if the event is found to be vulnerable. The normal nodes are not perceiving the ${\text{W}}_{{{\text{message}}}}$ in the ${\text{D}}_{0}$ region. The processing of ${\text{W}}_{{{\text{message}}}}$ in this scenario is summarized below:
  - When the malicious vehicle detects the ${\text{W}}_{{{\text{message}}}}$, it checks for ${\text{E}}_{{{\text{trust}}}}$ value.
  - If ${\text{E}}_{{{\text{trust}}}} = 1,$ it sets the value of ${\text{Trust}}$ to 0 with the probability of ${\text{P}}_{{{\text{MN}}}}$
  - Otherwise, it sets the value of ${\text{Trust}}$ to 1 having the probability of ${\text{P}}_{{{\text{MN}}}}$
  - Then the ${\text{M}}_{{{\text{alert}}}}$ is broadcasted to all nodes in ${\text{D}}_{{{\text{zone}}}}$.

Thus, the proposed trust-based localization benefits the VCPS by providing reliability, robustness, scalability and security.

## Results and discussion

In this section, the proposed trust-based localization performance for the vehicular network is evaluated with malicious vehicles. An enhanced vision on the performance of the suggested model is carried out using the NS2 (Network Simulator 2) simulator. The simulation parameters are listed in Table 1.

**Table 1:** Simulation parameters considered for the study.

Parameters	Values
Simulation speed (S)	1500
Transient speed (S)	300
Maximum speed (km/h)	60
No. of. vehicles	30
Duration of event (S)	50
% of attacker nodes	20%

**Figure 6:**
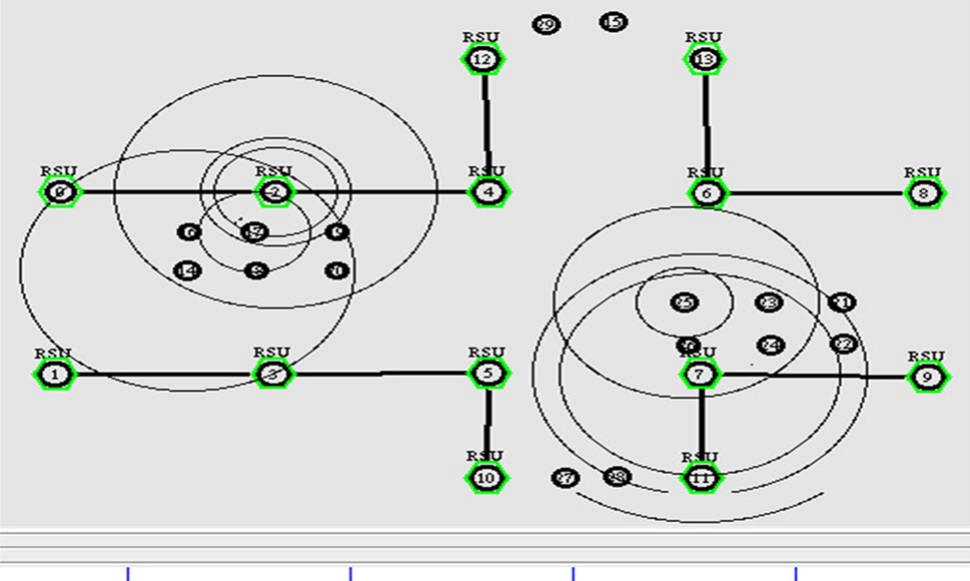
Broadcasting of location information.

The resiliency of GPS outage and location error in the network is achieved by pertaining the geometric localization with Extended Kalman Filter. The simulation is done with the increase in number of vehicles. It is prominently done for assessing the link failure with respect to the number of vehicles. Initially, the nodes start transmitting the location information for the predefined time T. Figure 6 depicts the initial simulation set up of the underlying scenario.

**Figure 7:**
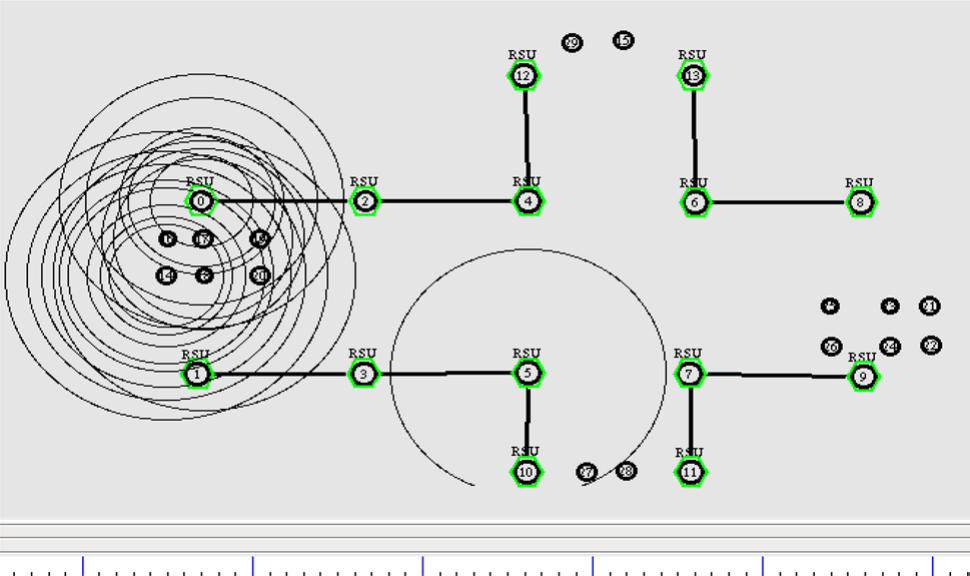
Exchanging future location of the vehicle.

Next, the nodes are exchanging the future location of the preceding value among the neighbors in the transmission range as shown in Fig. 7. In this stage, the throughput of the vehicle determined by the successful transmission of packets from source to the destination end. It can be calculated by the Eq. (31).

31 $${\text{Throughput}} = \frac{{\mathop \sum \nolimits_{{{\text{i}} = 1}}^{20} ({\text{N}}^{{\text{p}}} - {\text{N}}^{{\text{l}}} )}}{20}$$

**Figure 8:**
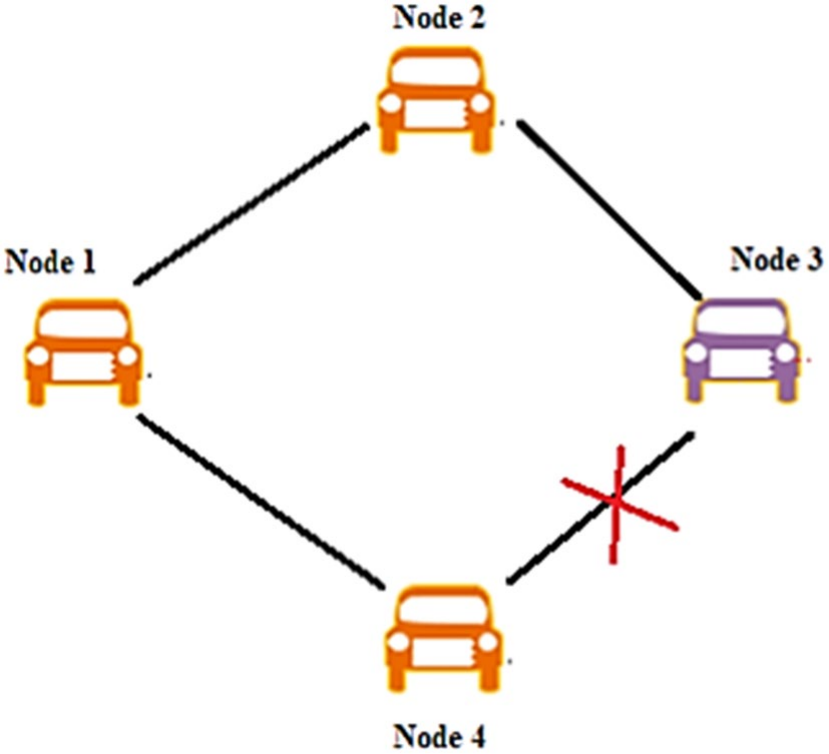
Simple attack scenario.

To evaluate the security performance of the proposed trust-based model, three attacker models have been chosen. First, the nodes are posed to a simple attack. Here, the malicious node misleads the neighboring vehicles by disrupts the communication rather than modifying the messages. The scenario of a simple attack is shown in Fig. 8. Here, the node 1 sends the data to the node 2, and node 2 sends the received data to node 3 consecutively. Node 3 is the attacker node which stops sending the message further to node 4.

**Figure 9:**
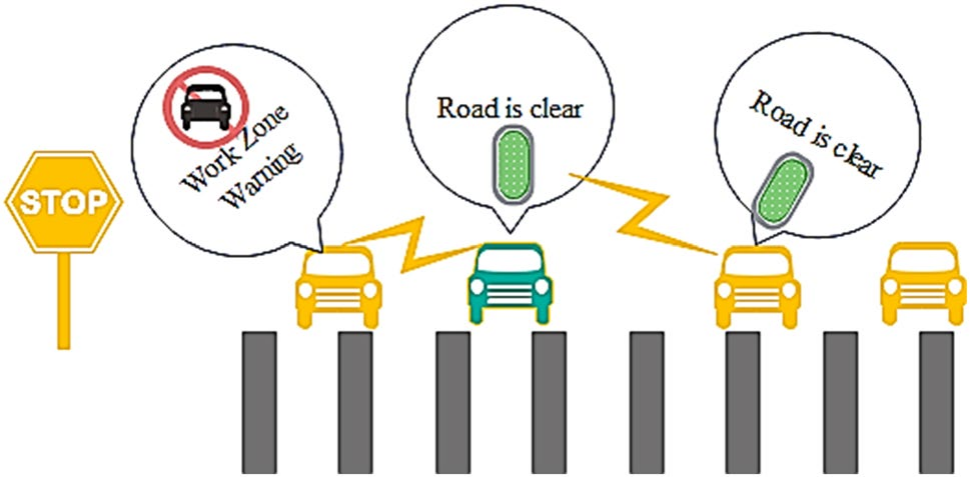
Bad mouth attack scenario.

Secondly, a bad-mouth attack is considered. Here, the attackers allure the network with false information by altering the patterns, making the nodes harder to compute the trust values. In this attack, the attackers share negative feedback on the victim node for the false computation of trust values. It makes the network puzzling and sometimes fatal. In Fig. 9, the first node tries to send a warning message to the successive nodes. Still, the malicious node 2 modifies the statement and sends the falsified data to the other vehicles to make the system compromised.

**Figure 10:**
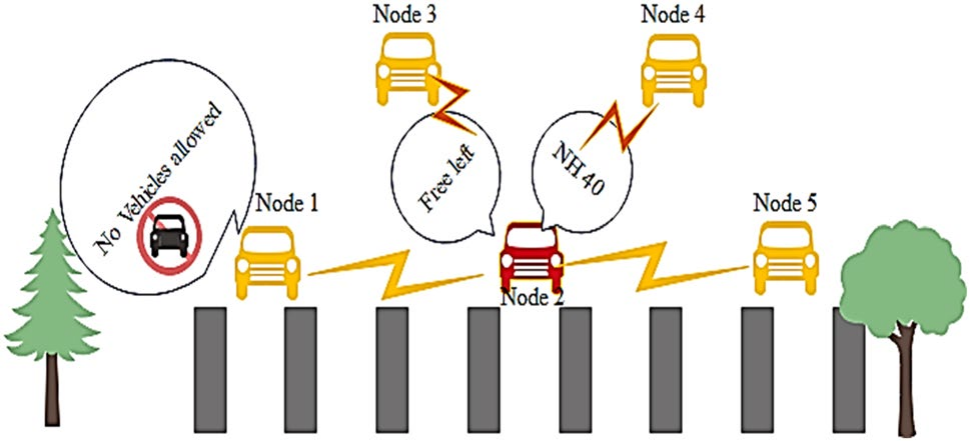
Zig- Zag attack model.

Third, the attacks which compromise the whole system by transmitting different information to different vehicles named Zig-Zag attack is considered. It deliberately conducts malicious behavior at various ends simultaneously. These attackers make the trust computation harder and inconsistent due to insufficient shreds of evidence on the attacker node. The attack scenario is given in Fig. 10. Here, Node 2 is malicious, sending different tampered data to multiple ends.

**Figure 11:**
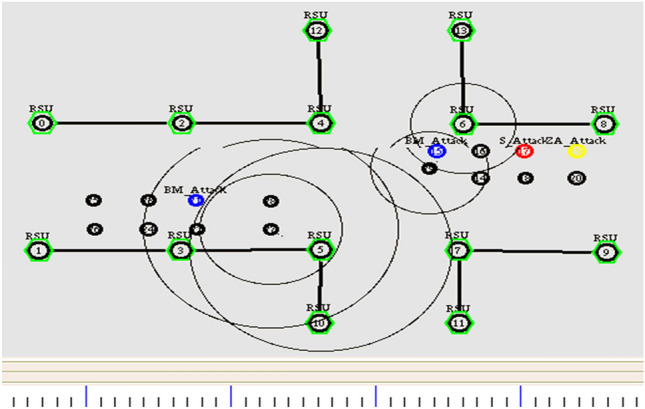
Attacker injection in vehicular network.

The simulation setup has tested the trust model with the presence of the attacks, as mentioned above. Figure 11 illustrates the scenario of three attacks in the simulated environment.

**Figure 12:**
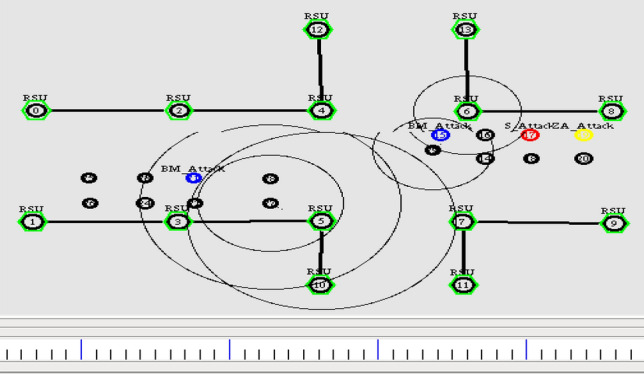
Malicious node added to list.

**Figure 13:**
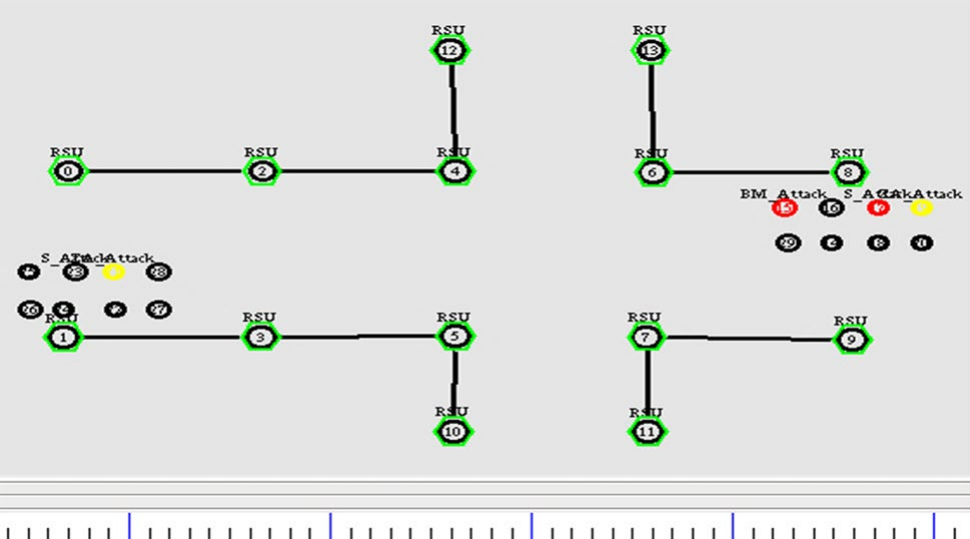
Establishment of trusted path.

Figure 11 shows that the yellow color node is a malicious vehicle launching Zig Zag attack, the blue color node indicates the bad mouth attack, and the red color node represents the simple attack. The malicious nodes start injecting the attack with its functionality among other nodes in the network. The trust model efficiently alters the path when receiving Event trust value as 0. Also, it isolates the nodes, and at an instance, it will not allow the malicious node to participate in the network. Figures 12 and 13 illustrates the node activity after receiving a negative trust value for malicious nodes. Meanwhile, the malicious node information is updated in the list. The trusted nodes start broadcasting the warning messages to all the other nodes in the network.

**Figure 14:**
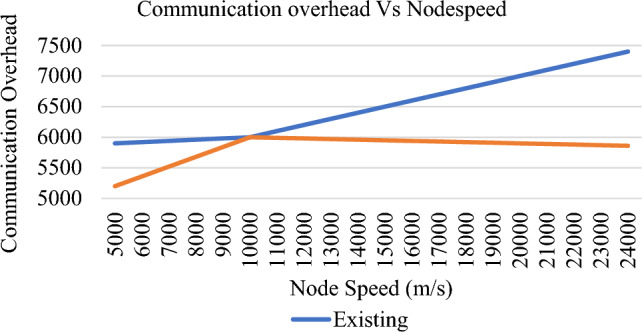
Communication overhead Vs Node speed.

The nodes start disseminating the data by selecting the trusted path excluding the malicious node as shown in Fig. 13. To evaluate the performance of proposed trust model, the following parameters have been considered and various graphs are plotted. For instance, Communication overhead, Precision rate and recall rate are taken for performance evaluation. Existing trust- based scheme TMHC^28^ is considered in this paper to analyze the performance of the proposed scheme. Communication overhead is the amount of time taken by the nodes to route the data from source to the destination. From Fig. 14, it is evident that the proposed scheme consumes less time for transferring the data from source end to destination.

**Figure 15:**
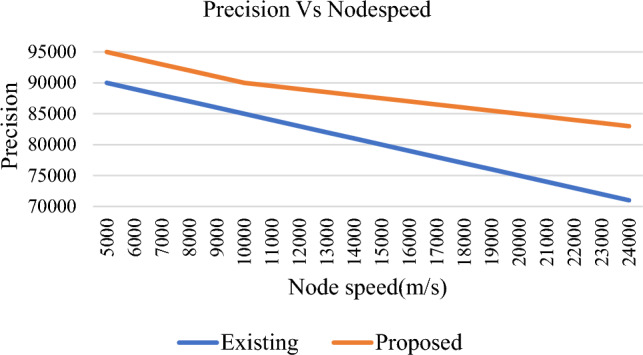
Precision Vs Node speed.

**Figure 16:**
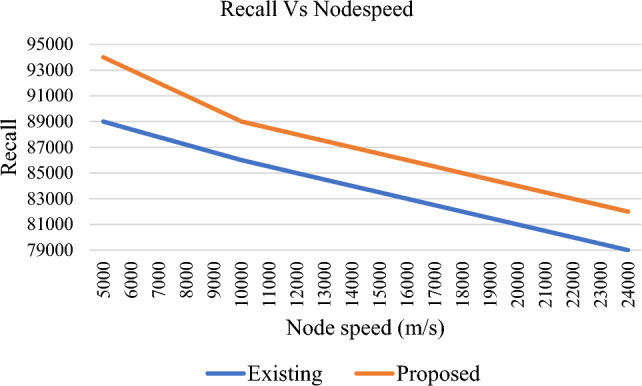
Recall Vs Node speed.

Precision rate is an important factor of every vehicular networks. It is the ratio of total number of successful interactions to the sum of successful and unsuccessful transmissions. The proposed trust model exhibits a better precision rate when compared to TMHC as shown in Fig. 15. Recall rate is the ratio of the number of successful communications recorded to the number of unsuccessful interactions. From Fig. 16, it is clear that the proposed scheme exhibits a higher precision rate when compared to TMHC.

## Conclusion

A novel trust based geometric localization technique is proposed in this paper. Many vehicular networks are faced with GPS outages and location errors induced by external factors. Hence, in this paper, a geometric localization technique is designed to perform the measurement of location of vehicles in case of GPS outages. It works on the present and historical information of the vehicles. Later, location prediction and correction technique are designed with Extended Kalman Filter where, the errors predicted and corrected by tuning the error covariance matrix to minimum. Furthermore, a trust-based security mechanism is explored in which the trusted and malicious nodes are accurately predicted. Using NS2, the proposed model is experimented with nodes deployed in a region. The proposed system is tested for performance by injecting Zig-Zag attack, Simple attack and Bad mouth attack into the network region. The trusted nodes efficiently detect the malicious nodes and starts establishing a secure path. Comparison graphs have revealed that the proposed system reports with a optimal communication overhead, precision and recall rate when compared with existing security models.

## Data Availability

The data used to support the findings of this study are included in the article.
